# Thallium group poisoning incident in Japan 2011

**DOI:** 10.1186/cc12207

**Published:** 2013-03-19

**Authors:** Y Namba, R Suzuki, J Sasaki, M Takayasu, K Watanabe, D Kenji, M Hayashi, Y Kitamura, M Kawamo, H Masaki, E Kyuuno, M Hayashi, M Yamaguchi, A Maeda

**Affiliations:** 1 Showa University Fujigaoka Hospital, Yokohama, Japan

## Introduction

Thallium is an odorless, tasteless, heavy metal that has been often used for intentional poisonings. In severe patients, thallium poisoning produces neuromuscular symptoms such as extreme pain and muscle weakness.

## Methods

Five case reports.

## Results

All patients worked at a pharmaceutical factory. They joined a tea party held at their workplace at the end of April 2011. The five patients drank tea from a teapot someone had put thallium in. A few days later, they complained of femoral numbness and pain caused by pressure. About a week later, three of five patients had profound hair loss. Three weeks after the party, they came to our ER. We thought that their symptoms might be caused by some chemicals. We searched the keywords: 'lower extremity pain', 'hair loss' and 'poison' in the Internet. As a result, thallium, mercury, lead, and so forth, were suspicious metals. In those metals, thallium was most likely because it was used in their factory. We immediately examined the blood concentration of several metals and ordered iron(III)hexacyanoferrate(II) that is known as the antidote for thallium poisoning. Only thallium was positive in the blood metal concentration test. Three patients consented to oral administration of an antidote. Two patients rejected administration because their symptoms were mild and getting better. All symptoms of all patients gradually disappeared by August. We also followed up the course of blood concentration of thallium. The concentration in three patients who took the antidote was reduced more rapidly than the two patients who did not take it.

## Conclusion

All patients recovered without any sequelae. Three patients' hair started to grow 3 months from ingestion of thallium, and after half a year their hair was restored to their former state. We had difficulty ordering iron(III)hexacyanoferrate(II) because this is also known as an antidote for cesium. On 11 March 2011 a megathrust earthquake and tsunami hit Japan and the giant tsunami gave rise to an accident at a nuclear power generation plant. Because the rumor of radioactive substances including cesium might be spread was the talk in the city near the nuclear power plant, the authorities put the antidote under heavy supervision. We could also collect the data for the course of thallium concentration. Thallium concentration of the patients who had an antidote was reduced more rapidly but these patients had a loose stool, thought to be a side effect of this antidote.
